# Racial/ethnic variations in gestational weight gain: a population-based study in Ontario

**DOI:** 10.17269/s41997-019-00250-z

**Published:** 2019-08-26

**Authors:** Yanfang Guo, Qun Miao, Tianhua Huang, Deshayne B. Fell, Alysha L. J. Harvey, Shi Wu Wen, Mark Walker, Laura Gaudet

**Affiliations:** 1 Better Outcomes Registry & Network Ontario, CHEO Research Institute - Centre for Practice-Changing Research Building, Ottawa, Ontario Canada; 2 Children’s Hospital of Eastern Ontario Research Institute, Ottawa, Ontario Canada; 3 Genetics Program, North York General Hospital, Toronto, Ontario Canada; 4 School of Epidemiology and Public Health, University of Ottawa, Ottawa, Ontario Canada; 5 OMNI Research Group, Clinical Epidemiology Program, Ottawa Hospital Research Institute, Centre for Practice-Changing Research, Ottawa, ON Canada; 6 Department of Obstetrics and Gynecology, University of Ottawa Faculty of Medicine, Ottawa, Ontario Canada

**Keywords:** Race/ethnicity, Weight gain, Body mass index, Pregnancy, Race/appartenance ethnique, Prise de poids, Indice de masse corporelle, Grossesse

## Abstract

**Objective:**

To explore inadequate and excessive gestational weight gain (GWG) among pregnant women of different racial/ethnic backgrounds in Ontario, Canada.

**Methods:**

A population-based retrospective cohort study was conducted among women who had prenatal screening and had a singleton birth in an Ontario hospital between April 2016 and March 2017. We estimated adjusted risk ratios (aRR) of racial/ethnic differences for inadequate or excessive GWG using multinomial logistic regression models. Interaction effects were examined to determine whether racial/ethnic difference in GWG varied by pre-pregnancy body mass index (BMI).

**Results:**

Among 74,424 women, the prevalence of inadequate GWG in White, Asian, and Black women was 15.7%, 25.8%, and 25.0%, and excessive GWG was 62.8%, 45.5%, and 54.7%, respectively. There were significant interaction effects between race/ethnicity and pre-pregnancy BMI for inadequate GWG (Wald *p* < 0.01) and excessive GWG (Wald *p* < 0.01). Compared with White women, Asian women had higher risk of inadequate GWG and lower risk of excessive GWG in all weight classes, and Black women had higher risk of inadequate GWG and lower risk of excessive GWG if their BMI was normal, overweight, or obese.

**Conclusion:**

Variations in unhealthy GWG by pre-pregnancy weight classes among Ontario White, Asian and Black women were observed. Individualized counseling regarding appropriate GWG is universally recommended. Additional consideration of racial/ethnic variations by maternal weight classes may help to promote healthy GWG in Canada.

**Electronic supplementary material:**

The online version of this article (10.17269/s41997-019-00250-z) contains supplementary material, which is available to authorized users.

## Résumé

**Objectif:**

Explorer la prise de poids gestationnelle (PPG) insuffisante et excessive chez les femmes enceintes de différentes origines raciales/ethniques en Ontario (Canada).

**Méthode:**

Une étude de cohorte populationnelle rétrospective a été menée auprès de femmes ayant subi un dépistage prénatal et accouché d’un enfant unique dans un hôpital de l’Ontario entre avril 2016 et mars 2017. Nous avons estimé le risque relatif ajusté (RRa) des différences raciales/ethniques dans la PPG insuffisante ou excessive à l’aide d’un modèle de régression logistique multinomiale. Nous avons examiné les effets d’interaction pour déterminer si les différences raciales/ethniques dans la PPG variaient selon l’indice de masse corporelle (IMC) d’avant la grossesse.

**Résultats:**

Chez 74 424 femmes, la prévalence de la PPG insuffisante chez les femmes blanches, asiatiques et noires était respectivement de 15,7 %, 25,8 % et 25,0 %, et celle de la PPG excessive était respectivement de 62,8 %, 45,5 % et 54,7 %. Il y a eu des effets d’interaction significatifs entre la race/l’appartenance ethnique et l’IMC d’avant la grossesse pour la PPG insuffisante (Wald *p* < 0,01) et la PPG excessive (Wald *p* < 0,01). Comparativement aux femmes blanches, les femmes asiatiques de toutes catégories de poids présentaient un risque supérieur de PPG insuffisante et un moindre risque de PPG excessive, et les femmes noires de poids normal, en surpoids ou obèses selon leur IMC présentaient un risque accru de PPG insuffisante et un moindre risque de PPG excessive.

**Conclusion:**

Des écarts ont été observés dans la PPG malsaine par catégorie de poids prégestationnel entre des femmes blanches, asiatiques et noires en Ontario. Des conseils personnalisés au sujet de la PPG appropriée sont recommandés dans tous les cas. Une attention supplémentaire aux différences raciales/ethniques par catégorie de poids maternel pourrait favoriser une PPG saine au Canada.

## Introduction

Inadequate and excessive gestational weight gain (GWG) have both been linked with a number of adverse maternal and neonatal outcomes, which in turn also vary by race/ethnicity (Headen et al. 2012). The United States (US) Institute of Medicine (IOM) developed GWG guidelines in 1990 and updated them in 2009, and these were adopted by Health Canada in 2010 (Health Canada 2010). In Canada, more than half of women exceed the 2009 national guidelines for weight gain during pregnancy, and approximately one fifth of women gain below the recommendations (Dzakpasu et al. 2015; Kowal et al. 2012), but data on racial/ethnic differences in GWG and their impact on adverse pregnancy outcomes were very limited. Exploring racial/ethnic variations in GWG is the first step to understand this issue in Canada.

Previous studies have reported racial/ethnic differences in GWG, with visible minorities at increased risk of inadequate GWG and lower risk of excessive GWG (Headen et al. 2015; Kinnunen et al. 2016; Pawlak et al. 2015; Bahadoer et al. 2015). A recent systemic review on GWG across three continents and diverse ethnicities indicated women in the United States and Europe have higher prevalence of GWG above guidelines and lower rates of GWG below guidelines than women in Asia (Goldstein et al. 2018). However, knowledge is limited in several ways. First, most of the population-based studies on racial/ethnic differences in GWG were conducted in the USA or Europe, and only two small studies have been performed in Canada (Kowal et al. 2012; Larouche et al. 2010). One study of 960 pregnant women from Montreal compared the GWG in six ethnic groups: White, Black, Latin American, East Asian, West Asian/Arab, and South Asian. No statistically significant differences were found between visible minorities and White women (Larouche et al. 2010). Kowal et al. used Canadian Maternity Experiences Survey (MES) data to describe GWG among women from several backgrounds, including Aboriginal, British Isles or French, European, and North American; however, Asian and Black populations were not examined (Kowal et al. 2012). Considering limitations of small sample size and selective participation bias in these studies, a larger population-based Canadian study is needed (Kowal et al. 2012; Larouche et al. 2010). Second, although Canada and the USA share some social and economic similarities, results of studies conducted in the USA may not directly apply to Canada due to differing racial/ethnic composition of the population and context. In the USA, 13.3% of the total population identify as Black and 5.7% identify as Asian (US Census Bureau 2016). In Canada, only 3.5% of the total population identify as Black, and 17.7% identify as Asian which comprise the largest and fastest-growing visible minority group in Canada (Statistics Canada 2017). Moreover, the majority of Black Canadians trace their family’s arrival to sometime after 1960, while more than 85% of Black Americans trace their ancestry back three or more generations in the USA (Vickers and Annette 2012; Attewell et al. 2010). As a consequence of immigration history, visible minority groups in Canada have increased gradually in recent decades. According to the 2016 Census, visible minorities now account for 22.3% of the total Canadian population and comprise 29.3% of Ontario’s total population (Statistics Canada 2017). The two largest visible minority groups are Asian and Black, each with a population exceeding one million in Canada (Statistics Canada 2017). Third, many studies used separate binary logistic regression models or one multinomial regression model to generate odds ratios of inadequate and excessive GWG (Liu et al. 2014; McDonald and Beyene 2015). Few studies used multinomial regression models to estimate the risk ratio (RR) of unhealthy GWG, which will avoid multiple testing problems and have more precise and accurate estimates, thus leading to correct conclusions (McDonald and Beyene 2015; Camey et al. 2014). Fourth, limited studies considered interaction effects between race/ethnicity and pre-pregnancy body mass index (BMI) on GWG, although studies indicated racial/ethnic disparities in pre-pregnancy BMI (Headen et al. 2015; Liu et al. 2014).

Ontario, the most populous province in Canada, has a high level of racial/ethnic diversity, providing a unique opportunity to examine racial/ethnic variations in GWG in Canada. The purpose of our study was to examine racial/ethnic differences in GWG in Ontario using data from a population-based birth registry (Dunn et al. 2011).

## Materials and methods

### Study design and data sources

In this population-based retrospective cohort study, we used data obtained from the Better Outcomes Registry & Network (BORN) Ontario birth registry. The BORN registry contains maternal demographic characteristics including race/ethnicity and clinical information related to pregnancy, including obstetrical complications and gestational weight gain. It has been assessed as a reliable, high-quality, comprehensive source of perinatal information covering 100% of hospital deliveries in Ontario (Dunn et al. 2011; BORN Ontario. BORN Data Quality Report 2012-2014 – Executive Summary n.d.). The prenatal screening program within the BORN registry routinely collects maternal racial/ethnic information to modify screening algorithms. We obtained information on socio-economic status, including neighbourhood household income and education quintiles by linking the birth registry with 2011 Census data by maternal residence postal code.

### Study population

We restricted our study population to women who had prenatal screening during pregnancies that resulted in a singleton birth in any Ontario hospital between April 1, 2016 and March 31, 2017. Approximately 70% of pregnant women received prenatal screening in Ontario in 2016 (BORN Ontario. Data Analysis for Annual Report 2014-2016. 2016 n.d.). Women who underwent prenatal screening were more likely to live in an urban area, receive care from an obstetrician, have a higher income, and have immigrant or refugee status (Hayeems et al. 2015). Women with any of the following conditions were excluded: gestational age at birth < 22 weeks or > 42 weeks, maternal age < 19 years old, multiple pregnancies, and lethal fetal anomalies. Only the first birth was included for those women who had two births during the study year. Women with missing, mixed, or other racial/ethnic background other than White, Asian, and Black were also excluded. We further limited to pregnancies with complete and plausible data on GWG and BMI: BMI range of 15–70 kg/m^2^ and GWG range of − 30–50 kg (McDonald et al. 2018).

### Measures

#### Primary outcome

The primary outcome was total GWG, expressed as a categorical variable (inadequate GWG, adequate GWG, and excessive GWG) classified according to pre-pregnancy BMI category. Actual total GWG was the difference between maternal weight at delivery and pre-pregnancy weight as recorded in the BORN database. Total GWG recommendations from the IOM 2009 guidelines (which were adopted by Health Canada in 2010) were used to define inadequate, adequate, and excessive GWG (Table 1) (Health Canada 2010). Because GWG is associated with gestational length, we accounted for the duration of gestation in our calculations of expected GWG. Expected GWG was calculated based on IOM recommendations for the amount of weight gain during the first trimester, which varied by pre-pregnancy BMI (underweight, 2 kg; normal weight, 2 kg; overweight, 1 kg; obese, 0.5 kg) (Headen et al. 2015; Rasmussen and Yaktine 2009) and weight gain during the second and third trimester. The expected GWG was then calculated as the recommended first trimester gain + (gestational age − 13) × (weight gain during the second and third trimesters). We calculated the ratio of actual GWG to the expected GWG according to 2009 IOM recommendations. If the ratio fell into the recommended range, then the woman was classified as adequate GWG group. If the ratio fell above or below these ranges, then total GWG was considered to be excessive or inadequate, respectively (Table 1) (Liu et al. 2014).

**Table 1:** 2009 Institute of Medicine (IOM) total GWG recommendations for singleton pregnancy

Pre-pregnancy BMI (kg/m^2^)	Recommended first trimester weight gain	Weekly recommended gain in 2nd and 3rd trimester	Recommended total GWG (kg) for full-term gestational age	Recommended ranges of expected weight gain based on the recommendation^a^
	Mean in kg	Mean (range) in kg/week	Range in kg	
Underweight, BMI < 18.5	2	0.51 (0.44–0.58)	12.5–18.0	0.79–1.14
Normal weight, 18.5 ≤ BMI < 25	2	0.42 (0.35–0.50)	11.5–16.0	0.86–1.20
Overweight, 25 ≤ BMI < 30	1	0.28 (0.23–0.33)	7.0–11.5	0.81–1.34
Obese, BMI ≥ 30	0.5	0.22 (0.17–0.27)	5.0–9.0	0.78–1.41

^a^The ranges were used as the basis for the following categories of weight gain adequacy: inadequate (less than the lower cutoff of recommendations), adequate (within recommended range), or excessive (greater than the upper cutoff of recommendations). The recommended range is calculated by dividing the lower and upper limits of the recommended weight gain range by the expected weight gain at 40-week gestation for each BMI group. For example, for underweight women, the range of weight gain is 12.5–18 kg, and the expected weight gain is 15.77 kg (2 kg + [40–13] × 0.51). Thus, the adequate range of expected weight gain based on the recommendation for underweight women is 0.79–1.14, where 0.79 = (12.5/15.77) and 1.14 = (18/15.77)

#### Exposure

Women’s race/ethnicity (White, Asian, and Black) was our main independent variable of interest, which was self-reported and recorded by the prenatal care provider who completed the prenatal screening requisition.

#### Main covariate and modifier

Pre-pregnancy BMI, calculated by dividing self-reported pre-pregnancy weight (kg) by self-reported height (m), squared, was the main covariate and modifier. We used BMI categories based on the World Health Organization (WHO) standards: underweight (BMI < 18.5 kg/m^2^), normal weight (18.5 ≤ BMI < 25 kg/m^2^), overweight (25 ≤ BMI < 30 kg/m^2^), or obese (BMI ≥ 30 kg/m^2^) (World Health Organization n.d.).

#### Other covariates

We also included a wide range of potential confounders for unhealthy GWG, including pre-pregnancy BMI categories (underweight, normal, overweight, or obese), mother’s age at birth (≤ 24, 25–29, 30–34, or ≥ 35 years), neighbourhood household median income quintile (lowest, 2nd, 3rd, 4th, highest), neighbourhood education quintile (percentage of adults 26 to 64 years having a university degree), parity (nulliparous or multiparous), conception type (in vitro fertilization, intrauterine insemination, or no assisted reproductive technology), smoking during pregnancy (yes or no), pre-existing maternal health conditions (chronic hypertension, diabetes, chronic heart disease, pulmonary disease [yes or no]), and antenatal health care provider (inclusive of family physician, obstetrician, family physician and obstetrician, midwife, other, or none).

### Statistical analysis

Maternal demographic characteristics and clinical factors were compared among the three racial/ethnic groups. We described continuous variables by mean ± standard deviation (SD) and categorical variables by count and percent (%). We assessed the associations between covariates and race/ethnicity using analysis of variance or Kruskal-Wallis H tests for continuous data and chi-square tests for categorical data.

Prevalence of inadequate and excessive GWG stratified by pre-pregnancy BMI categories was examined among White, Asian, and Black women. Multinomial logistic regression models were used to estimate the adjusted risk ratio (aRR) with 95% confidence intervals (CI) of inadequate or excessive GWG across race/ethnicity groups, with White women as the reference (SAS Institute Inc 2016). We first obtained the model parameter estimates and GWG probabilities for each race/ethnicity from a statement of PROC LOGISTIC and then calculated the RR and 95% CI by using the NLEstimate macro (SAS Institute Inc 2016). Potential confounders were identified by comparing the measure of association before and after adjusting for confounders. If the difference between the two measures of association was 15% or more, the confounder was adjusted in the multivariate model. Even though adjustment for socio-economic status (SES) has been considered a form of overadjustment on causal intermediates and leads to biased estimates of the total effect in some ethnicity studies, we still adjusted for SES in models as it is difficult to remove confounding effects of SES when investigating racial/ethnic disparities in health outcomes. We used multiple imputation methods to account for missing data on the following covariates: neighbourhood household income (6.3% missing), education (5.6% missing), parity (0.6% missing), and antenatal health care provider (2.0% missing). Ten datasets were imputed by using the fully conditional specification (FCG) logistic regression method. We also conducted a sensitivity analysis to compare our main results using imputed data, with a complete case analysis. Interaction effects were also tested to determine whether racial/ethnic differences in GWG varied by pre-pregnancy weight category. We used Wald tests to assess the significance of interaction for both inadequate and excessive GWG at the *p* ≤ 0.10 level. If significant interaction was detected, we reported the magnitude of racial difference within each stratum of pre-pregnancy weight class. All analyses were performed using the Statistical Analysis System (SAS) for Windows, version 9.4 (SAS Institute, Cary, NC), with 2-tailed tests and a significance level of *p* < 0.05.

## Results

**Fig. 1:**
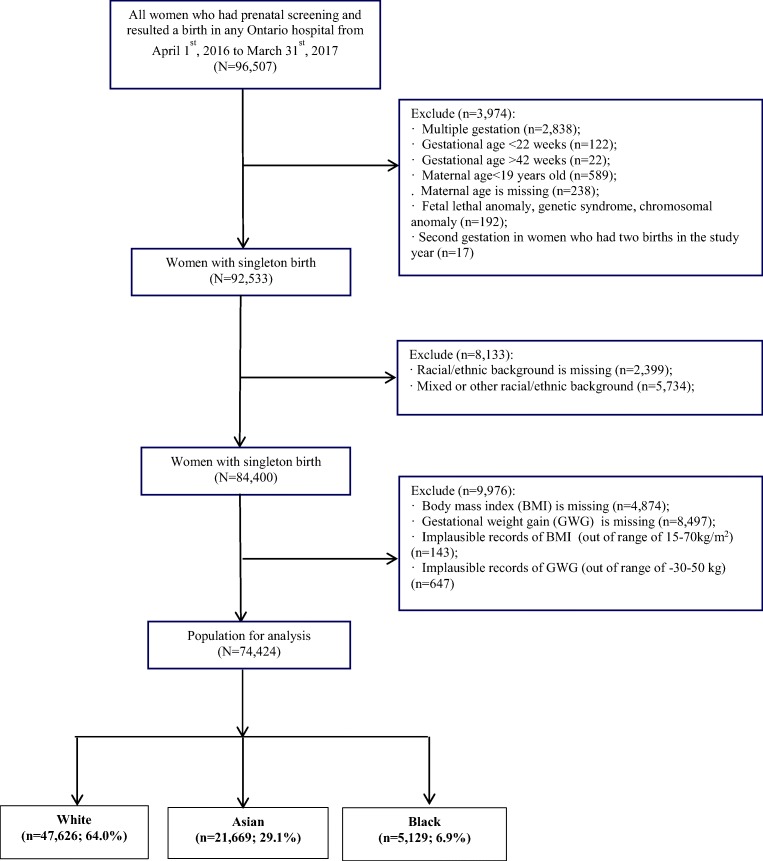
Flow chart of study population

Following exclusions, a total of 74,424 women were included in the study. Of these, 64.0% were Whites, 29.1% were Asians, and 6.9% were Blacks (Fig. 1). Table 2 shows the distribution of selected characteristics by race/ethnicity. Compared with White women, Asian women were older, less likely to be obese, more educated, less likely to smoke during pregnancy, and less likely to have chronic disease. Compared with White women, Black women were more likely to be obese, more likely to reside in a neighbourhood in the lowest income quintile, and less likely to smoke during pregnancy.

**Table 2:** Characteristics of women who were eligible for the study by race/ethnicity characteristics

Characteristics	Total		White		Asian		Black		*p*
	*N*	%	*n*	%	*n*	%	*n*	%	
N	74,424	100.0	47,626	63.9	21,669	29.1	5129	6.9	
Pre-pregnancy BMI (kg/m^2^) (mean ± SD)	25.1 ± 5.7		25.7 ± 6.0		23.4 ± 4.5		27.1 ± 6.1		< 0.001
Underweight (BMI < 18.5)	3903	5.9	1839	4.3	1882	9.8	182	4.0	< 0.001
Normal weight (BMI 18.5–24.9)	34,873	52.4	21,620	50.6	11,522	60.0	1731	37.6	
Overweight (BMI 25.0–29.9)	16,142	24.3	10,500	24.6	4225	22.0	1417	30.8	
Obese (BMI ≥ 30)	11,605	17.4	8761	20.5	1575	8.2	1269	27.6	
Maternal age at delivery (years) (mean ± SD)	31.4 ± 4.8		31.1 ± 4.8		32.1 ± 4.5		31.0 ± 5.6		
≤ 24	5701	8.6	4171	9.8	873	4.5	657	14.3	< 0.001
25–29	17,099	25.7	11,221	26.3	4698	24.5	1180	25.7	
30–34	26,581	40.0	17,138	40.1	7967	41.5	1476	32.1	
≥ 35	17,142	25.8	10,190	23.9	5666	29.5	1286	28.0	
Neighbourhood household median income quintile									
Quintile 1 (lowest)	14,319	21.5	7679	18.0	4590	23.9	2050	44.6	< 0.001
Quintile 2	11,587	17.4	7604	17.8	3188	16.6	795	17.3	
Quintile 3	12,740	19.2	8411	19.7	3652	19.0	677	14.7	
Quintile 4	16,231	24.4	10,715	25.1	4805	25.0	711	15.5	
Quintile 5 (highest)	11,646	17.5	8311	19.5	2969	15.5	366	8.0	
Missing	4681	6.3	2774	5.8	1631	7.5	276	5.4	
Neighbourhood education quintile (percentage of university degrees among adults 25–64 years old)									
Quintile 1 (lowest)	8884	13.4	7472	17.5	791	4.1	621	13.5	< 0.001
Quintile 2	12,069	18.1	8944	20.9	2072	10.8	1053	22.9	
Quintile 3	15,077	22.7	9058	21.2	4720	24.6	1299	28.2	
Quintile 4	17,400	26.2	9690	22.7	6617	34.5	1093	23.8	
Quintile 5 (highest)	13,093	19.7	7556	17.7	5004	26.1	533	11.6	
Missing	4190	5.6	2442	5.1	1503	6.9	245	4.8	
Parity									
Nulliparous	29,512	44.4	19,771	46.3	8098	42.2	1643	35.7	< 0.001
Multiparous	37,011	55.6	22,949	53.7	11,106	57.8	2956	64.3	
Missing	431	0.6	285	0.6	111	0.5	35	0.7	
Conception type									
In vitro fertilization	1454	2.2	1028	2.4	373	1.9	53	1.2	< 0.001
Intrauterine insemination	1265	1.9	919	2.2	309	1.6	37	0.8	
No assisted reproductive technology	63,804	95.9	40,773	95.4	18,522	96.4	4509	98.0	
Smoking during pregnancy									
Yes	4825	7.3	4435	10.4	200	1.0	190	4.1	< 0.001
No	61,698	92.7	38,285	89.6	19,004	99.0	4409	95.9	
Maternal pre-existing health conditions (chronic hypertension, diabetes, chronic heart disease, pulmonary disease)									
Yes	4310	6.5	3368	7.9	632	3.3	310	6.7	< 0.001
No	62,213	93.5	39,352	92.1	18,572	96.7	4289	93.3	
Antenatal health care provider									
Inclusive of family physician	5483	8.2	4309	10.1	899	4.7	275	6.0	< 0.001
Obstetrician	40,907	61.5	21,943	51.4	15,569	81.1	3395	73.8	
Family physician + obstetrician	9623	14.5	8075	18.9	1201	6.3	347	7.5	
Midwife	8460	12.7	6965	16.3	1074	5.6	421	9.2	
None	78	0.1	43	0.1	27	0.1	8	0.2	
Other	626	0.9	519	1.2	81	0.4	26	0.6	
Missing/unknown	1,346	2.0	866	2.0	353	1.8	127	2.8	
Gestational age at delivery (weeks) (mean ± SD)	38.8 ± 1.8		38.9 ± 1.8		38.7 ± 1.8		38.6 ± 2.2		< 0.001
< 37 weeks	3919	5.9	2429	5.7	1161	6.0	329	7.2	< 0.001
≥ 37 weeks	62,604	94.1	40,291	94.3	18,043	94.0	4270	92.8	

Note: Missing data of neighbourhood household median income and education quintile, parity, and antenatal health care provider were excluded from the percentage calculation

Table 3 shows the distribution of inadequate and excessive GWG across the three racial/ethnic groups. Overall, 19.3% of women had inadequate GWG and more than half (57.2%) of women had excessive GWG. The prevalence of inadequate GWG was higher in Asian (25.8%) and Black women (25.0%) than in White women (15.7%). Conversely, Asian women and Black women had a lower prevalence of excessive GWG (45.5% and 54.7%) than White women (62.8%).

**Table 3:** Distribution of GWG among pregnant women by race/ethnicity, stratified by pre-pregnancy BMI categories

Outcome	Total		White		Asian		Black	
	*N*	%	*n*	%	*n*	%	*n*	%
Overall women								
Inadequate GWG	14,367	19.3	7494	15.7	5590	25.8	1283	25.0
Adequate GWG	17,504	23.5	10,245	21.5	6216	28.7	1043	20.3
Excessive GWG	42,553	57.2	29,887	62.8	9863	45.5	2803	54.7
Underweight								
Inadequate GWG	1678	38.0	723	34.7	875	41.0	80	40.4
Adequate GWG	1424	32.2	643	30.8	730	34.2	51	25.8
Excessive GWG	1318	29.8	720	34.5	531	24.9	67	33.8
Normal weight								
Inadequate GWG	8282	21.1	4139	17.0	3582	27.6	561	28.9
Adequate GWG	12,367	31.5	7364	30.3	4445	34.2	558	28.7
Excessive GWG	18,633	47.4	12,837	52.7	4972	38.2	824	42.4
Overweight								
Inadequate GWG	2182	12.2	1069	9.2	812	17.1	301	19.0
Adequate GWG	2120	11.8	1095	9.4	788	16.6	237	14.9
Excessive GWG	13,641	76.0	9438	81.3	3155	66.4	1048	66.1
Obese								
Inadequate GWG	2225	17.4	1563	16.3	321	18.0	341	24.3
Adequate GWG	1593	12.5	1143	11.9	253	14.2	197	14.1
Excessive GWG	8961	70.1	6892	71.8	1205	67.7	864	61.6

*GWG*, gestational weight gain; *BMI*, body mass index

Table 4 shows the aRR of unhealthy gestational weight gain among three race/ethnicity groups. Compared with White women, after adjusting for maternal demographic and clinical characteristics, the overall aRRs of inadequate GWG were 1.20 (95% CI, 1.18, 1.22) and 1.29 (95% CI, 1.24, 1.33) for Asian and Black women, respectively, and aRRs of excessive GWG were 0.72 (95% CI, 0.71, 0.73) and 0.83 (95% CI, 0.80, 0.86) for Asian and Black women, respectively.

**Table 4:** Adjusted RR for racial/ethnic differences in risk of unhealthy GWG, stratified by pre-pregnancy BMI categories

	Adjusted RR (95% CI)				
	All women^a^	Underweight^b^	Normal weight^b^	Overweight^b^	Obese^b^
Inadequate GWG vs. adequate GWG					
Asian	1.20 (1.18, 1.22)	1.18 (1.12, 1.22)	1.40 (1.38, 1.45)	1.37 (1.32, 1.43)	1.07 (1.02, 1.11)
Black	1.29 (1.24, 1.33)	1.08 (0.98, 1.18)	1.45 (1.41, 1.53)	1.53 (1.46, 1.60)	1.27 (1.22, 1.34)
White	1.00	1.00	1.00	1.00	1.00
Excessive GWG vs. adequate GWG					
Asian	0.72 (0.71, 0.73)	0.81 (0.79, 0.84)	0.83 (0.81, 0.84)	0.89 (0.88, 0.90)	0.95 (0.94, 0.97)
Black	0.83 (0.80, 0.86)	1.04 (0.98, 1.11)	0.72 (0.67, 0.78)	0.88 (0.87, 0.89)	0.90 (0.88, 0.91)
White	1.00	1.00	1.00	1.00	1.00

*RR*, risk ratio; *95% CI*, 95% confidence intervals; *GWG*, gestational weight gain; *BMI*, body mass index

Multinomial logistic regression models were used to estimate the risk ratios.

Multiple imputation methods were used to impute missing values of covariates. Missing values of median household income, education level, parity, and antenatal care provider were imputed by fully conditional specification (FCS) logistic regression method.

^a^Models were adjusted for pre-pregnancy BMI, maternal age, maternal neighbourhood household median income level, neighbourhood education level, parity, conception type, smoking during pregnancy, maternal pre-existing health problem, and antenatal health care provider

^b^Models were adjusted for maternal age, maternal neighbourhood household median income level, neighbourhood education level, parity, conception type, smoking during pregnancy, maternal pre-existing health problem, and antenatal health care provider

There were significant interaction effects between race/ethnicity and pre-pregnancy BMI for inadequate GWG (Wald *p* < 0.01) and excessive GWG (Wald *p* < 0.01). Thus, stratified results by pre-pregnancy BMI were shown in Table 4 as well. Compared with White women, Asian women with all weight classes had higher risk of inadequate GWG (underweight, aRR 1.18, 95% CI [1.12, 1.22]; normal, aRR 1.40, 95% CI [1.38, 1.45]; overweight, aRR 1.37, 95% CI [1.32, 1.43]; obese, aRR 1.07, 95% CI [1.02, 1.11]) and lower risk of excessive GWG (underweight, aRR 0.81, 95% CI [0.79, 0.84]; normal, aRR 0.83, 95% CI [0.81, 0.84]; overweight, aRR 0.89, 95% CI [0.88, 0.90]; obese, aRR 0.95, 95% CI [0.94, 0.97]). Compared with Whites, Black women with BMI ≥ 18.5 kg/m^2^ had higher risk of inadequate GWG (normal, aRR 1.45, 95% CI [1.41, 1.53]; overweight, aRR 1.53, 95% CI [1.46, 1.60]; obese, aRR 1.27, 95% CI [1.22, 1.34]) and lower risk of excessive GWG (normal, aRR 0.72, 95% CI [0.67, 0.78]; overweight, aRR 0.88, 95% CI [0.87, 0.89]; obese, aRR 0.90, 95% CI [0.88, 0.91]). Our sensitivity analysis showed that point estimates using multiple imputation for missing covariates were similar to our complete cases results (Supplementary Table 1).

## Discussion

Our study, based on a large multi-ethnic cohort, found that GWG varied among White, Asian, and Black women by pre-pregnancy BMI, even after accounting for the difference in baseline characteristics of the groups. Compared with White women, Asian women regardless of their pre-pregnancy weight classes and Black women except underweight had higher risk of inadequate gestational weight gain and lower risk of excessive gestational weight gain. Excessive GWG was, however, an important issue for all racial/ethnic groups. Although minority women appeared to gain less weight than White women, they were still not protected from excessive GWG. We also observed significant modification effects of race/ethnicity and pre-pregnancy BMI groups on inadequate GWG and excessive GWG in this study.

To our knowledge, this is the first population-based study in Canada that examines racial/ethnic differences in GWG among White, Asian, and Black women. The overall prevalence of inadequate and excessive GWG observed in our study were consistent with those from a Canadian surveillance report, which showed that approximately 20% of women have inadequate GWG and more than 50% of women have excessive GWG (Dzakpasu et al. 2015; Kowal et al. 2012). Studies from the USA indicate that the prevalence of unhealthy GWG varies among non-Hispanic White, non-Hispanic Black, and Hispanic women, with Hispanic and Black women being more likely to have inadequate GWG and less likely to have excessive GWG compared with White women (Headen et al. 2012; Pawlak et al. 2015; Liu et al. 2014; De Jongh et al. 2014; Vanstone et al. 2017; Mendez et al. 2016; Hunt et al. 2013). However, in the US studies, Asian women were combined with other racial/ethnic groups or were removed from analysis due to the small population (Headen et al. 2012; Pawlak et al. 2015; Liu et al. 2014; De Jongh et al. 2014; Vanstone et al. 2017; Mendez et al. 2016; Hunt et al. 2013). With a relatively high proportion of visible minority populations in Ontario, particularly for Asian women, our study provides further evidence on the differences between GWG in White and Asian women—Asian women in Ontario are more likely to have inadequate GWG and less likely to gain excessive weight during pregnancy than White women, regardless of weight classes. Our findings contradict those from a small study conducted in Montreal in a tertiary care centre, which compared GWG within six racial/ethnic groups and found no significant differences between visible minorities and White women in GWG (Larouche et al. 2010). The inconsistency between our study and the Montreal study is most likely related to the lack of study power in the Montreal study, which only included 960 women in total, fewer than 400 of whom represented ethnic minorities (Larouche et al. 2010).

An important contribution of this study is its more appropriate method of estimating the RR by using a multinomial logistic regression model (SAS Institute Inc 2016). Instead of using separate binary logistic regression models to generate odds ratios of inadequate and excessive GWG (each compared with adequate GWG), we used one multinomial logistic regression model, which can overcome other drawbacks of separate binary models, such as redundancy and loss of information that result when only a subset of the data is analyzed at a time, as well as multiple testing problems that arise from analyzing several pairs of categories (McDonald and Beyene 2015). Moreover, directly estimating the risk ratios for common outcomes (> 10%) is less biased than odds ratios (Camey et al. 2014). To our knowledge, multinomial logistic regression model does not provide RR directly, but the NLEstimate macro using the fitted model information saved with the STORE statement in PROC LOGISTIC could produce RR and 95% CI (SAS Institute Inc 2016). In addition, our large sample size provided sufficient power to test the interaction between race/ethnicity and pre-pregnancy BMI on unhealthy GWG, and our findings of interaction effects were consistent with several other large studies (Headen et al. 2015; Camey et al. 2014; Fontaine et al. 2012; Hickey et al. 1999), but differ from the results from two smaller studies (Pawlak et al. 2015; Caulfield et al. 1996).

There were several limitations of our study. Pre-pregnancy weight, weight at delivery, and racial/ethnic information were all self-reported. Although high concordance has been observed between self-reported and clinically recorded weight as well as between birth certificate data and clinically recorded GWG (Natamba et al. 2016; Holland et al. 2013; Bannon et al. 2017), studies using measured weight are needed to address these limitations and confirm our study findings. Goldstein et al. indicated variations in GWG between Asian and women in the USA and Europe were diminished when regional specific BMI categories were used (Goldstein et al. 2018). However, it is unclear how well the IOM guidelines suit different racial/ethnic groups as we used the WHO guidelines to identify BMI categories. Misreporting race/ethnicity may occur as well. Moreover, unavailability of GWG by trimester limited our ability to examine the effect by gestational periods. We were unable to investigate the differences in GWG between South Asian and East Asian women due to the lack of specific identity information for these two racial/ethnic groups in the database. Differential findings from East Asia compared with those from South Asia have been reported for both obesity and GWG (Kinnunen et al. 2016; Yi et al. 2015). In addition, selection bias may have resulted from exclusion of women who did not complete prenatal screening, but the direction and magnitude are uncertain. Finally, although adjustment was conducted for a range of potential confounders, residual confounding may still exist.

## Conclusion

Our large population-based study revealed variations in maternal gestational weight gain among White, Asian, and Black women in Canada. In terms of racial difference, we found Asian women in all pre-pregnancy weight classes and Black women if their BMI was normal, overweight, or obese had higher risk of inadequate GWG and lower risk of excessive GWG than White women. Further work should examine whether the racial/ethnic differences in pre-pregnancy GWG by trimester and whether racial/ethnic differences in GWG lead to differences in maternal and childhood outcomes. Individualized counseling regarding appropriate GWG is currently universally recommended in Canada. Additional consideration of racial/ethnic variations may help promote healthy pregnancy-related weight gain.

## Electronic supplementary material

ESM 1 (DOCX 15 kb)
