# Supplementary material for: Racial/ethnic variations in gestational weight gain: a population-based study in Ontario
Source: Can J Public Health. 2019 Aug 26;110(5):657–67. doi: 10.17269/s41997-019-00250-z (PMC6825031; doi:10.17269/s41997-019-00250-z)
Supplement: Supplementary file 1 — (DOCX 15 kb) [file 41997_2019_250_MOESM1_ESM.docx]

**Supplementary Table 1. Adjusted RR for racial/ethnic differences in risk of unhealthy GWG, stratified by pre-pregnancy BMI categories (complete cases)**

|  | **Adjusted RR (95% CI)** | | | | |
| --- | --- | --- | --- | --- | --- |
|  | **All women ^a^** | **Underweight ^b^** | **Normal weight ^b^** | **Overweight ^b^** | **Obese ^b^** |
| ***Inadequate GWG vs. adequate GWG*** | | | | | |
| Asian | 1.30 (1.25, 1.35) | 1.21 (1.15, 1.32) | 1.50 (1.41, 1.75) | 1.65 (1.41, 1.88) | 1.17 (1.12, 1.21) |
| Black | 1.35 (1.31, 1.38) | 1.06 (0.86, 1.26) | 1.65 (1.51, 1.83) | 1.71 (1.56, 1.90) | 1.47 (1.35, 1.64) |
| White | 1.00 | 1.00 | 1.00 | 1.00 | 1.00 |
|  |  |  |  |  |  |
| ***Excessive GWG vs. adequate GWG*** | | | | | |
| Asian | 0. 66 (0.64, 0.69) | 0.76 (0.62, 0.89) | 0.78 (0.75, 0.84) | 0.91 (0.88, 0.94) | 0.96 (0.94, 0.97) |
| Black | 0.77 (0.72, 0.82) | 1.01 (0.88, 1.18) | 0.88 (0.82, 0.94) | 0.90 (0.88, 0.92) | 0.94 (0.92, 0.98) |
| White | 1.00 | 1.00 | 1.00 | 1.00 | 1.00 |

*RR: risk ratio, 95% CI: 95% confidence interval, GWG: gestational weight gain, BMI: body mass index*

*1. Multinomial logistic regression models were used to estimate the risk ratio.*

*a. Models were adjusted for pre-pregnancy BMI, maternal age, maternal neighbourhood household median income level, neighbourhood education level, parity, conception type, smoking during pregnancy, maternal pre-existing health problem and antenatal health care provider.*

*b. Models were adjusted for maternal age, maternal neighbourhood household median income level, neighbourhood education level, parity, conception type, smoking during pregnancy, maternal pre-existing health problem and antenatal health care provider.*
